# Advances in Modeling Multiple Myeloma Within the Bone Marrow Tumor Microenvironment for Exploration of Current and Emerging Therapies

**DOI:** 10.3390/cancers18132050

**Published:** 2026-06-24

**Authors:** Charlotte E. J. Toomes, Oliver G. Best, Timothy Hollenberg, Rose Turner, Claudine S. Bonder, Barbara J. McClure

**Affiliations:** 1 Flinders Health and Medical Research Institute, College of Medicine and Public Health, Flinders University, Bedford Park, SA 5042, Australia; 2 Department of Haematology, Flinders Medical Centre, Bedford Park, SA 5042, Australia; 3 Centre for Cancer Biology, College of Health, Adelaide University, Adelaide, SA 5000, Australia; claudine.bonder@adelaide.edu.au

**Keywords:** multiple myeloma, bone marrow tumor microenvironment, 2D models, 3D models, organ-on-a-chip

## Abstract

Multiple Myeloma (MM) is a hematological malignancy characterized by the clonal proliferation and survival of neoplastic plasma cells (PCs) within the bone marrow (BM), where disease progression is critically supported by interactions with the BM tumor microenvironment (TME). Despite significant advances in therapeutic strategies, MM remains incurable, underscoring the need for improved preclinical models to better understand the disease biology and therapeutic response. This review summarizes current and emerging MM treatment approaches and critically examines the development of models designed to more accurately recapitulate interactions between MM-PCs and the surrounding BM niche. We describe established and emerging modeling platforms, with emphasis on advanced three-dimensional (3D) culture systems and highlight their unique contributions to the preclinical assessment of both existing and novel therapies. The advantages of 3D models, including in vitro and in silico systems, over traditional two-dimensional (2D) models are discussed, alongside a comparative evaluation of scaffold-free and scaffold-based approaches. In addition, the benefits and recent advances in the customization of BM niche simulation using microfluidic technologies and organ-on-a-chip platforms are reviewed. The application of 3D models in MM research is increasingly enabling the study of disease pathogenesis, progression, drug resistance and precision-medicine approaches (informed by biomarker discovery). Although standardized preclinical approaches for evaluating MM therapeutics are currently lacking, the growing imperative to reduce reliance on preclinical animal models highlights the importance of alternate systems. Consequently, the development and adoption of physiologically relevant models that accurately recapitulate MM-PC interactions with the BM TME will be critical for advancing future therapeutic strategies in MM.

## Simple Summary

Multiple myeloma is a blood cancer of plasma cells that develops in the bone marrow, where interactions with the surrounding microenvironment play a central role in disease progression and resistance to therapy. Despite advances in treatment, the disease remains incurable, highlighting the need for improved experimental models that more closely recapitulate human disease. This review discusses recent advances in three-dimensional and bioengineered platforms that better mimic the bone marrow microenvironment. These models provide a more physiologically relevant setting to study disease biology, treatment response, and drug resistance. Advancing these technologies may improve preclinical drug screening and toxicity testing, facilitate the identification of new therapeutic targets, and ultimately accelerate the development of more effective therapies for patients with multiple myeloma.

## 1. Background

**Figure 1:**
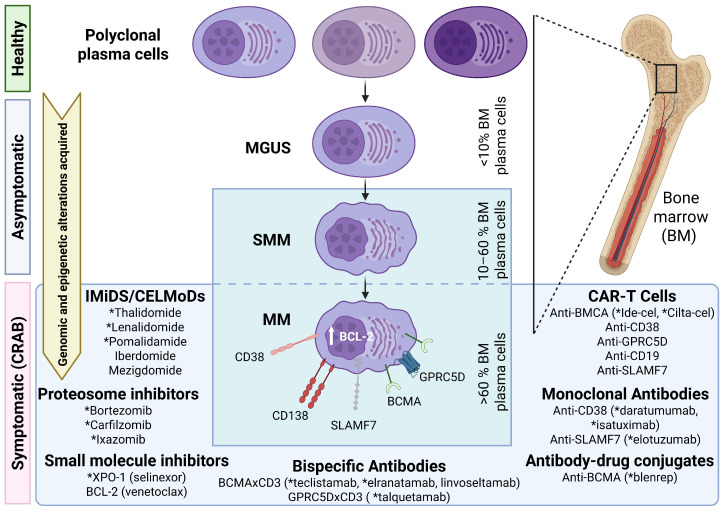
Multiple myeloma disease progression and current and emerging therapeutics. Multiple myeloma disease progression from asymptomatic MGUS and SMM to symptomatic MM: Summary of current and emerging treatment approaches for active MM, which include immunomodulatory drugs, proteasome inhibitors, small-molecule inhibitors, monoclonal and bi-specific antibodies, antibody–drug conjugates and CAR T-cells. Examples of each are listed and those with FDA approval denoted with an asterisk (*). For a full list (including chemotherapeutics), see Table 1. Abbreviations: MGUS: monoclonal gammopathy of undetermined significance, SMM: smoldering multiple myeloma, MM: multiple myeloma, CD38: cluster of differentiation 38, CD138: cluster of differentiation 138, SLAM7: SLAM Family Member 7, BCMA: B-cell maturation antigen, GPRC5D: G-protein-coupled receptor, class C, group 5, member D, IMiDs: immunomodulatory drugs, CELMoDs: cereblon E3 ligase modulators, XPO1: exportin 1, BCL-2: B-cell leukemia/lymphoma 2, CD3: cluster of differentiation 3, CD19: cluster of differentiation 19, BM: bone marrow, CRAB: calcium, renal impairment, anemia, bone lesions. Created in BioRender. Bonder, C. (2026) https://BioRender.com/9q8uhim.

Multiple myeloma (MM) is the second most common blood cancer, and currently, 188,000 new cases are diagnosed annually worldwide [1]. MM is characterized by the uncontrolled proliferation of neoplastic plasma cells (PCs), predominantly in the bone marrow (BM), which secrete non-functional monoclonal immunoglobulins and together result in osteolytic bone lesions, anemia, and systemic end-organ damage, including renal impairment (Figure 1). The recent introduction of new treatment strategies, including immunomodulatory drugs (IMiDs), proteasome inhibitors (PIs) and monoclonal antibodies (mAbs) has improved survival rates over the last two decades; however, the 5-year survival rate remains poor at 50% with a median overall survival (OS) of 5.5 years [2]. Some patients now achieve long-term survival, particularly those with standard-risk features and low tumor burden; however, relapse remains inevitable, and MM is still considered incurable. MM generally affects older individuals, and the incidence of MM is increasing, particularly in men, those above 50 years of age, and those living in high-income countries [2,3,4], potentially as a result of improved diagnostics combined with an aging population. Outcomes remain particularly poor for older patients, with 5-year progression-free survival rates (PFSs) remaining as low as 20% [5], without the same improvement in survival rates observed in those under 60 years [2]. The persistence of MM-PCs and disease progression is exquisitely dependent on the BM tumor microenvironment (TME) [6]. Here, we aim to review MM and the advances in modeling this disease within the BM TME in emerging multi-dimensional in vitro systems. Given the advantages the BM TME provides for both MM-PC survival and treatment resistance, these new models recapitulate the in vivo interactions between MM-PCs and the BM TME and improve our understanding of MM biology. This will allow for optimal preclinical assessment of emerging therapies and aid in improving the successful translation of new therapeutic approaches to the clinic.

## 2. MM Disease Progression, Diagnosis, and Staging Overview

MM develops through a multistep process in which healthy PCs progressively acquire genomic and epigenetic alterations that ultimately lead to the malignant transformation and clonal expansion of MM-PCs [7]. Normal PCs are terminally differentiated B-cells that are generally non-proliferative and secrete antigen-specific immunoglobulins as part of the adaptive immune system. Conversely, most malignant MM-PCs secrete a non-functional immunoglobulin, called paraprotein (or M-protein), and a serum free light chain (sFLC), a hallmark feature of the monoclonal gammopathy group of hematolymphoid malignancies [7]. MM is preceded by a non-malignant condition called monoclonal gammopathy of undetermined significance (MGUS), an asymptomatic condition in which the BM has <10% PCs, and levels of serum M-protein are <30 g/L [8]. MGUS is usually discovered as an incidental finding during other clinical investigations and often remains stable for many years. As neoplastic PCs acquire additional alterations and mutations, the condition progresses to smoldering multiple myeloma (SMM), defined by BM clonal MM-PCs between 10–60% or serum M-protein >30 g/L, and the absence of any myeloma-defining events [9]. The rate of progression from MGUS to MM is 1% per year [9], while the rate of progression from SMM to MM is significantly higher at around 10% per year during the first five years [9]. A diagnosis of MM is not typically made until clinical manifestations of end-organ pathologies are present, defined by the CRAB criteria: hypercalcemia (C), renal impairment (R), anemia (A) and osteolytic bone lesions (B) [10]. In 2014 this was updated (SLiM CRAB) to define the presence of defined biomarkers of disease including a clonal BM PC percentage ≥60%, an involved:uninvolved sFLC ratio ≥ 100 or >1 focal lesion detected by magnetic resonance imaging (MRI) to confirm a myeloma diagnosis.

The underlying biological heterogeneity of MM leads to diverse outcomes in response to standard treatments. The ability to accurately stratify patients according to risk has improved in recent years with the use of a scoring system based on diagnostic and clinical parameters. Stratification is used to help determine appropriate treatment timing and regimen [9]. Approximately 20% of newly diagnosed MM (NDMM) patients have disease classified as high risk (HR). Since 2015, the most widely used staging system for identifying HR MM patients at diagnosis has been the Revised International Staging System (R-ISS). The R-ISS was introduced by the International Myeloma Working Group (IMWG) [11], updated from the original ISS devised in 2005 [12]. The R-ISS classifies disease into three grades (R-ISS-I, II and III), with R-ISS-III identifying HR disease based on standard risk (SR) ISS criteria, elevated levels of serum β2-microglobulin and lactate dehydrogenase (LDH), and the presence of HR chromosomal abnormalities (CAs). HR CAs include deletion of a region of chromosome 17 (del(17p)) that encompasses tumor protein p53 (*TP53*) and translocations involving chromosomes 4, 14 and 16 (t(4; 14) or t(14; 16)) [11]. The R-ISS staging system was updated again in 2022 to the second revised (R2)-ISS [13]. Both the R-ISS and R2-ISS consider the presence of CAs and reflect the genomic heterogeneity of the disease. Results from the implementation of the R2-ISS, outside of clinical trials, demonstrate the ability of the scoring system to predict HR patients who require more aggressive therapies [14]. Recent studies have also shown that a gain of chromosome 1 (1q+) is an indicator of HR disease, and that so-called double-hit patients with del(17p) accompanied by mutations in the remaining *TP53* allele, have significantly worse outcomes than patients with del(17p) without a *TP53* mutation [13,15]. While the presence of a monoallelic del(1p32) is associated with reduced survival rates, the outcome of patients with biallelic del(1p32) is significantly worse and is therefore considered an indication of ultra HR disease [16]. This Consensus Genomic Staging (CGC) of HR MM incorporated molecular/genomic factors that have emerged to provide a contemporary clinical staging for HR MM [17]. The further refinement of stratifying patients at diagnosis based on genomic features is evidenced by a recent study in which optical genome mapping was used to provide refined prognostic information [18]. The advances in artificial intelligence approaches that utilize multiomic datasets to refine risk in MM are increasingly improving risk stratification beyond those currently used clinically [19]. This will be beneficial for approximately 20% of patients with functionally high-risk disease who lack recognized high-risk features and are therefore not identified at diagnosis by current risk assessment strategies [20]. In light of these advancing techniques in genomic diagnosis and the rapidly evolving landscape of novel therapies, an updated consensus definition of HR myeloma was recently published by the IMWG that moves beyond the traditional prognostic markers of the R-ISS by incorporating clinical data with contemporary genomic data and risk stratification evidence [17].

## 3. Current Treatment Strategies Based on Disease Stratification

The most widely used Food and Drug Administration (FDA)-approved therapies for NDMM include proteasome inhibitors (PIs), immunomodulatory drugs (IMiDs), corticosteroids (dexamethasone and prednisone), and mAbs [9] (Figure 1). The introduction of these therapies has significantly improved outcomes for the majority of MM patients over the past few decades [5]. A summary of all FDA-approved therapies for the treatment of MM is provided in Table 1. The effects of currently approved PIs (bortezomib, carfilzomib and ixazomib) are mediated by accumulation of misfolded proteins in the endoplasmic reticulum (ER) within MM-PCs, leading to ER stress-induced cell apoptosis [21]. The IMiDs (thalidomide, lenalidomide and pomalidomide) target cereblon (CRBN), which modulates the substrate specificity of the CRBN-DDB1 (DNA damage-binding protein 1) E3 ubiquitin ligase complex, resulting in the proteasomal degradation of proteins essential for MM-PC survival [22]. In comparison, the mAbs used in the clinic bind specific antigens expressed by MM-PCs, including cluster of differentiation (CD) 38 (daratumumab and isatuximab) and SLAM Family Member 7 (SLAMF7) (elotuzumab). Upon binding, these mAbs induce MM-PC death through multiple mechanisms, including Fc-dependent effector functions, such as complement-dependent cytotoxicity (CDC), antibody-dependent cell-mediated cytotoxicity (ADCC), and antibody-dependent cellular phagocytosis (ADCP) [23]. The most widely used therapeutic regimen is a combination comprised of lenalidomide, bortezomib, and dexamethasone (VRd) (triplet therapy), with or without daratumumab (D-VRd) or isatuximab (Isa-VRd). These regimens are used either as frontline therapy or as an induction therapy prior to autologous stem cell transplant (ASCT), the latter of which remains the standard of care for transplant-eligible patients [9]. More traditional chemotherapy-based regimens incorporating alkylators (melphalan, cyclophosphamide and bendamustine), vinca alkaloids (vincristine), or anthracyclines (doxorubicin) are now used less frequently and predominantly in the setting of aggressive or treatment-resistant disease or when access to newer therapies is limited [9].

More recently, targeted therapies with bispecific antibodies (BiSAbs), chimeric antigen receptor (CAR) T-cell therapies, and a nuclear export (exportin 1, XPO1) inhibitor (selinexor) have gained FDA approval and are now increasingly available for clinical use in RRMM [9]. Inhibition of XPO1 by selinexor results in the accumulation of tumor suppressor proteins in the nucleus and prevents nuclear export of oncogenic mRNAs and their subsequent translation into oncogenic proteins [24]. Currently approved BiSAbs target CD3 on T-cells and specific antigens expressed by the MM-PCs, including B-cell maturation antigen (BCMA; CD269) (elranatamab and teclistamab) and G-protein-coupled receptor, class C, group 5, member D (GPRC5D) (talquetamab). Through their affinity for both cell types, these bi-specific T-cell engagers (BiTEs) activate T-cells in proximity to the MM-PCs, leading to antibody-dependent cellular toxicity and cell-mediated tumor cell death [25]. BiSAbs are currently FDA approved for the treatment of RRMM patients who have received four or more prior lines of therapy including a PI, IMiD, and anti-CD38 mAbs [26]. CAR T-cell therapies for MM involve isolation of T-cells and genetic engineering to express receptors that recognize specific antigens on the MM-PCs [27]. Currently approved CAR T-cell therapies (idecaptagene vicleucel and ciltacaptagene autoleucel) target BCMA, a protein which has long been of interest as a target for MM therapy as it is highly expressed on MM-PCs and not on healthy plasma or B-cells [28].

Other novel therapies in the RR setting include the antibody–drug conjugate (ADC) belantamab mafodotin (Blenrep), being the first of this class to be approved in the US and Europe for the treatment of RRMM. Although Blenrep showed efficacy in early trials, FDA approval was withdrawn in 2022 when a confirmatory trial failed to show superiority over standard of care. More recently, Blenrep was re-approved by the FDA given its “substantial efficacy”, and therefore, Blenrep remains a drug of interest. Additionally, the BH3-mimetic, B-cell leukemia/lymphoma 2 (BCL-2) inhibitor venetoclax is available through off-label use for a subset of RRMM patients who harbor a translocation between chromosomes 11 and 14 (t(11;14)) [29]. MM-PCs with t(11;14) have increased expression of *BCL-2* [30], which prompted trials of venetoclax for treatment of this HR subset of MM patients. Although venetoclax demonstrated promising clinical efficacy for t(11; 14) MM patients in early-phase trials, the FDA placed a hold on venetoclax for MM due to safety concerns, following the results of the phase III BELLINI trial, which demonstrated twice as many deaths among patients in the venetoclax group compared with placebo, despite deeper responses and better PFS rates [31]. However, this hold was soon lifted and while the subsequent CANOVA trial suggested venetoclax was associated with improved PFS duration, the results were not statistically significant [32].

Minimal residual disease (MRD) status has emerged as a critical indicator of treatment response and prognosis [33] and is becoming increasingly used in response-adapted treatment paradigms. As newer therapies such as BiSAbs and CAR T-cells achieve deeper responses, advanced techniques using next-generation flow cytometry (NGF), next-generation sequencing (NGS), digital droplet PCR (ddPCR), and mass spectrometry have been developed to enable more sensitive MRD assessment [33]. Although advances in treatment approaches have led to improved response rates and survival of MM patients over the last two decades [5], there remains a subset of MM patients with poor prognoses, for whom treatment options are limited. In particular, survival rates among patients over 80 years of age at diagnosis have remained extremely low over the past few decades [33]. Furthermore, the majority of patients will experience disease relapse, highlighting the need for ongoing research that includes development of novel therapeutic approaches and models that recapitulate the complexity of the disease within the BM niche.

## 4. Emerging Treatment Strategies for MM

A suite of other therapeutic agents are now available for MM patients through clinical trial programs. Trials of next-generation PIs (e.g., marizomib) and mAbs targeting GPRC5D, LAG-3 (lymphocyte-activation gene 3), PD-1 (programmed cell death protein 1), and MICA/MICB are currently underway. However, most of the trials focusing on NDMM and RRMM patients are of BiSAbs, tri-specific antibodies (TriSAbs), CAR T-cell therapies, ADCs, cereblon E3 ligase modulatory drugs (CELMoDs), and CAR natural killer (NK)-cell therapies. The BiSAbs and TriSAbs currently in development target T-cells via CD3, while TriSAbs also target MM-PCs via affinity for BCMA. Additional targets on MM-PCs that are being investigated in BiSAbs and TriSAbs constructs are CD38, GPRC5D, and Fc receptor-homolog 5 (FcRH5). Similar targets are being investigated in CAR T-cell therapies (e.g., anitocabtagene autoleucel and arlocabtagene autoleucel) including BCMA, GPRC5D, FcRH5, SLAMF7, CD19, and transmembrane activator and CAML interactor (TACI). Dual targeting CAR T-cell therapies are also under development, which have affinity for both BCMA and either CD19, TACI, GPRC5D, or SLAMF7.

A newer class of therapies being assessed for the treatment of MM are CELMoDs (e.g., iberdomide and mezigdomide), the next-generation IMiDs with additional phenyl and morpholino moieties which increase their affinity for CRBN and enhance their potency via increased degradation of IKAROS and Aiolos (IKZF3) [34]. ADCs continue to represent a promising therapeutic class in MM, with ongoing development targeting BCMA, CD38, and CD46. Also being investigated are small-molecule inhibitors that target BCL2, histone deacetylases (HDACs), key kinases, and myeloid cell leukemia 1 (MCL-1).

## 5. Emerging Novel Biomarkers in MM

Given the genomic heterogeneity among MM patients, a focus of ongoing research is to identify additional prognostic biomarkers that may also serve as targets for novel therapeutic approaches. Defining the role(s) that novel biomarkers play in the pathobiology of MM may address the urgent need for new treatment strategies.

A recent study reported that the cadherin desmoglein-2 (*DSG2*) was expressed in MM-PCs but not healthy PCs [35]. Gene expression of *DSG2* was significantly elevated in ~30% of newly diagnosed MM patients and identified a subset of patients with HR disease, independent of chromosomal abnormalities and other diagnostic markers [35]. Expression of several other genes has also been reported to correlate with prognosis in MM, including dual-specificity phosphatase (*DUSP1*) [36], tyrosine kinase *WEE1* [37] and mucin 1 (*MUC1*) and *XPO1* [38], while expression of Chitinase 3-like protein-1 (*CHI3L1*) [39] has been associated with disease progression. High levels of circulating cell-free tumor DNA at diagnosis have also been shown to correlate with disease burden and poor prognosis [40] and may provide a non-invasive means of stratifying patients and personalizing treatments according to disease genomics [41].

## 6. The MM BM Tumor Microenvironment (BM TME)

The BM is the primary site in which malignant PCs accumulate and proliferate in patients with MM. The BM TME plays a crucial role in the pathogenesis of MM by facilitating the interaction between MM-PCs and other BM-resident cells, including mesenchymal stem cells (MSCs), osteoclasts (OCs), vascular endothelial cells (ECs) and immune cells. Expression of adhesion molecules by MM-PCs and BM stromal cells, along with secretion of cytokines and growth factors, creates a supportive niche that promotes MM-PC survival and proliferation. Regions of the BM populated by MM-PCs are collectively referred to as the MM BM TME.

Following genomic alterations in lymph-node germinal centers, neoplastic PCs enter the circulation, where they migrate to the BM guided by chemotactic signals released from the sinusoidal vasculature [42]. The chemokine, stromal cell-derived factor-1α (SDF-1α or CXCL12), which is largely produced by BM-resident ECs, is particularly potent in recruiting CXC motif chemokine receptor 4 (CXCR4) expressing PCs to the BM [43,44]. Once inside the BM, PCs reside in niches and actively transform the local environment, creating an interactive ecosystem that supports their survival and proliferation [45]. Retention of PCs in the BM niche is facilitated by adhesion molecules such as integrins α4β1, α4β7, and CD44 that engage with vascular cell adhesion molecule-1 (VCAM-1, or CD106), mucosal vascular addressin cell adhesion molecule-1 (MAdCAM), fibronectin and hyaluronic acid (HA) [46,47]. The unique architecture of long bones means that the expansion of MM-PCs creates a niche in which oxygen availability is extremely low; the response of MM-PCs and ECs to this hypoxia triggers angiogenesis within the BM [48].

Bone is a highly vascularized tissue with distinct heterogeneous structures that receive ~10–15% of the resting cardiac output [49]. The vasculature within the bone is anatomically unique, comprised of three EC subtypes (Type E (CD31-high/endomucin-medium), Type H (CD31-medium/endomucin-high), and Type L (CD31-low/endomucin-low), all of which have sinusoidal characteristics. Type H ECs are closely aligned with the growth plate of long bones and engage with arteries/arterioles to facilitate PC entry into the marrow. Type L ECs are the predominant subtype in the adult BM [50] and engage with resident stromal cells, circulating immune cells and the extracellular matrix (ECM) (reviewed in Chen et al., 2021 [51]).

In addition to its vasculature, the MM BM TME is highly heterogeneous with dynamic interactions between cellular and non-cellular components that play a fundamental role in the development and progression of the disease. The cellular component of the BM niche is comprised of ECs, pericytes, MSCs, fibroblasts, OCs, osteoblasts (OBs), immune cells (myeloid cells and lymphocytes), sympathetic neurons and adipocytes. MM-PCs colonize regions in close proximity to OBs [52], which support their survival and dormancy, while OCs activate MM-PCs by remodeling the endosteal niche [53]. OBs and bone-resorbing OCs play a significant and interdependent role in bone metabolism. The interaction between α4β1-expressing MM-PCs and OBs that express VCAM-1 causes a reduction in Runt-related transcription factor 1 (RUNX1) activity, leading to a loss of OB function and the formation of osteolytic lesions [54]. Osteolytic lesions are further exacerbated by the translocation of β-catenin to the nucleus and inhibition of WNT signaling in OBs, due to the presence of the Dickkopf-1 (DKK1) protein, which is secreted by MSCs [55,56]. Myeloid cells are abundant in the BM TME [57], with M2 macrophages secreting pro-cancerous soluble factors, including vascular endothelial growth factor (VEGF), fibroblast growth factor 2 (FGF2), transforming growth factor beta (TGF-β), interleukin (IL)-10 and matrix metalloproteases (MMPs), which suppress T lymphocyte proliferation [58], and increase resistance to chemotherapy [57]. Tumor-associated macrophages have also been shown to protect MM-PCs through an interaction mediated by adhesion molecules, including PSGL-1:P-selectin and ICAM1:αLβ2 [59]. Increased levels of myeloid-derived suppressor cells (MDSCs) in the BM of MM patients promote the differentiation of regulatory T lymphocytes (Tregs) [60] and inhibit the expansion of cytotoxic T-cells [61]. The production of TGF-β and IL-10 by BM-infiltrating Tregs further inhibits effector T-cell proliferation and is associated with decreased survival rates among MM patients [62]. NK cells are also abundant in the BM TME of MM patients, but their cytotoxic function has been shown to be significantly impaired [63]. The survival and resistance of MM-PCs to chemotherapy is supported by the production of IL-6 and IL-10 by MSCs and T-regs, respectively, and interaction with cells via MM-PC expressed adhesion molecules α4β1 and CD44 [64,65,66]. IL-6 activates OCs, which in turn activate dormant MM-PCs in the BM niche. This process, along with the increased vascular permeability induced by IL-6, facilitates the dissemination of MM-PCs [67]. Platelets also play a significant role in MM by “cloaking” the MM-PCs, protecting them from recognition by immune cells and supporting their engraftment in the BM niche [68,69].

By inhibiting the proteasome, PIs induce an accumulation of proteins within MM-PCs, leading to cellular stress and apoptosis. However, it is now clear that PIs also have significant effects on the BM TME by inhibiting cell-to-cell interactions, angiogenesis and cytokine production, which collectively support the survival of MM-PCs in the BM (reviewed by Ito et al., 2020 [70]). The IMiDs lenalidomide and pomalidomide are also now widely used for the treatment of MM, while trials of the next-generation IMiD, mezigdomide, are currently underway (reviewed in Mo et al., 2025 [71]). Similar to PIs, IMiDs have multifaceted mechanisms of action not only exhibiting direct cytotoxic effects towards tumor cells but also modulating the interaction between MM-PCs, immune cells, and stromal cells within the BM TME. It is now recognized that the mechanism of action of IMiDs involves activation of T- and NK-cells, which increases their secretion of interferon-γ and IL-2. IMiDs also activate and promote the maturation of dendritic cells (DC), which enhances their presentation of antigens and ultimately improves the recognition and elimination of MM-PCs by cytotoxic T-cells. Furthermore, IMiDs are known to have anti-angiogenic effects that restrict the ability of MM-PCs to survive and proliferate in the BM TME (reviewed by Guo et al., 2022 [72]).

There is growing evidence that exosomes play a role in the pathobiology of MM. Exosomes are generated by intraluminal budding of vesicles from the late endosomal membrane of vascular ECs, MSCs, fibroblasts, OBs, OCs and immune cells and contain cargo (DNA, mRNA, miRNA, integrins, growth factors and enzymes) [73] that facilitates the progression of MM via many of the aforementioned cellular interactions and processes (reviewed in Angelica Ortiz, 2021 [74]). For example, exosomes derived from MM BM-MSCs facilitate dissemination of MM-PCs [75]. Exosomes can inhibit OB differentiation and induce OC formation and activity by triggering signaling via the AKT (protein kinase B) pathway. Exosomes create an immunosuppressive environment via effects on MDSCs, M2 macrophages, NK cells and their negative impact on CD8+ T-cells (reviewed in Menu and Vanderkerken, 2022 [76]). Exosomes likely represent a hitherto underappreciated mechanism of cell-to-cell communication that creates niches that support the expansion of MM-PCs, drive disease progression and confer drug resistance.

## 7. Modeling the MM BM TME

The BM TME plays a crucial role in driving MM-PC proliferation and resistance to therapy [77,78]. Consequently, modeling the MM BM TME is essential for investigating the pathobiology of the disease and for the preclinical evaluation of novel treatment approaches [79]. However, the complexity of the cell-to-cell interactions, the milieu of secreted factors, and the hypoxic conditions make it challenging to recreate the BM niche in the laboratory [80]. This is particularly true of in vitro studies, but also of in vivo preclinical studies which utilize animals that are either immunocompromised to enable human cancer cell engraftment or murine orthotopic models that may not directly translate to human disease. As previously discussed, the BM is comprised of a range of different cell types, including both hematopoietic stem cells (HSCs) and MSCs. HSCs give rise to cells of the myeloid and lymphoid lineages, while MSCs differentiate into a range of different cell types, including fibroblasts, OBs, and ECs. These cell types play important roles in normal hematopoiesis and in the BM TME of cancers, including MM. This section reviews the details of various in vitro models developed in the context of the MM BM TME.

**Figure 2:**
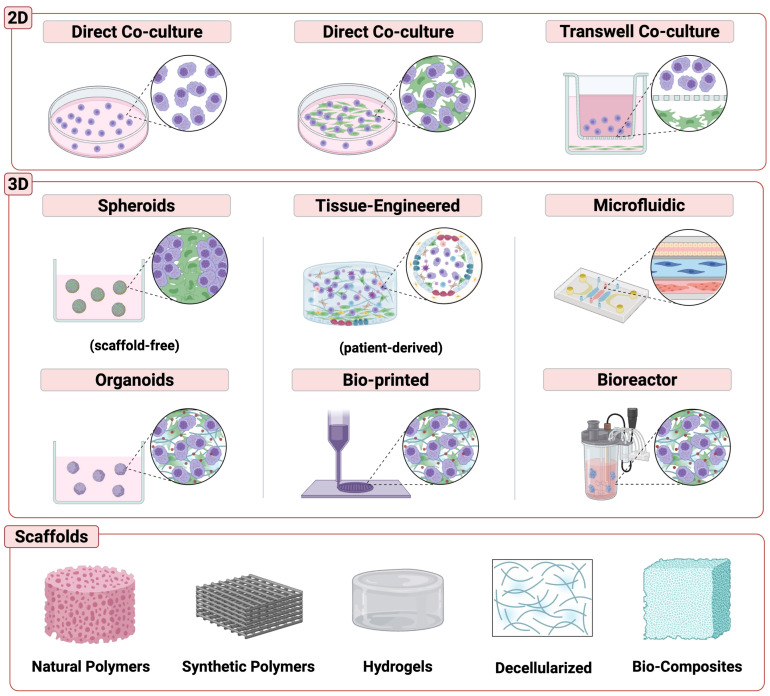
Evolution of in vitro modeling for recapitulating myeloma plasma cell and bone marrow tumor microenvironment. Created in BioRender. Toomes, C. (2026) https://www.BioRender.com/hyiudb6.

Two-dimensional (2D) monoculture models, where tumor cells are cultured on plastic surfaces under controlled conditions (Figure 2), have long been a staple in assessing tumor biology and responses to therapeutics [79]. However, these models fail to capture the complexity of the BM TME in vivo [81,82,83,84,85]. This is illustrated by evidence that 2D monoculture models are insufficient to support the long-term survival of primary MM patient cells and result in progressive changes in the morphology and phenotype of the cells [86,87]. To address these issues, multiple more complex models of the MM BM TME that incorporate aspects of the BM niche have been developed over the last few decades. Given the recent FDA Modernization Act 2.0 [88], the utilization of alternate models that accurately recapitulate the interaction between MM-PCs and the BM TME will be crucial for the transition away from animal models for preclinical studies of future MM therapies.

### 7.1. Two-Dimensional In Vitro Models of the MM BM TME

Two-dimensional (2D) models of the MM BM TME generally involve supplementation of culture medium with soluble factors or co-culture of MM-PCs on layers of “feeder” cells. A broad range of growth factors, cytokines, and chemokines that influence the proliferation and survival of MM-PCs have been identified [89]. IL-6 has been recognized as a crucial factor in the proliferation of MM-PCs [90,91], and supplementing culture medium with IL-6 has been shown to induce a more patient-like transcriptomic phenotype in a subset of MM cell lines [92], albeit at concentrations that far exceed those in MM patient plasma [93]. However, even at high concentrations, IL-6 alone is unable to maintain the survival of primary MM-PCs purified from the BM of patients with early-stage disease [89,94]. This suggests that while IL-6 plays a role, other soluble factors and cell–cell interactions are required to support the survival and proliferation of MM-PCs in the BM TME [89].

Co-culture models, where MM-PCs are cultured in 2D structures with other cell types that comprise the BM TME (Figure 2), have demonstrated that contact with MSCs [95,96,97,98,99], OBs [100], OCs [101], macrophages [57], and DCs [102] can support the survival and promote proliferation of primary MM-PCs and cell lines. As MSCs make up a very low proportion of the cells in the BM (0.001–0.01% of mononuclear cells; MNCs) [103,104], these cells are often expanded prior to use in co-culture with MM-PCs. Interestingly, MM-PCs can induce senescence of MSCs, reduce their ability to differentiate into OBs, and increase their differentiation into fibroblasts and adipocytes [105,106,107]. Furthermore, MSCs have been shown to promote disease progression and vascularization in in vivo MM models [106]. Co-culture with MM-PCs also increases the secretion of several cytokines and growth factors from MSCs, including IL-6, IL-10, tumor necrosis factor alpha (TNF-α), hepatocyte growth factor (HGF), and B-cell activating factor (BAFF), even for several weeks after removal of the MM-PCs, suggesting that the cell-to-cell interaction promotes autocrine signaling [108]. These findings are supported by transcriptomic, epigenetic, and microRNA expression studies of the MSCs following co-culture with MM-PCs, which demonstrated dysregulation of genes associated with tumor growth, drug resistance, angiogenesis, and bone remodeling, including C-X-C motif chemokine ligand 1 (*CXCL1*), IL-8, and angiopoietin-like 4 (*ANGPTL4*) [109,110,111,112,113,114,115,116,117]. Additionally, the transfer of non-coding RNA (e.g., microRNA) between MM-PCs and MSCs via extracellular vesicles can drive gene expression changes in both cell types [118,119,120,121,122,123,124]. These studies highlight the significance of the interaction between MM-PCs and other cell types that comprise the BM TME and suggest that MM-PCs can remodel the BM TME to promote their survival and proliferation.

### 7.2. In Silico Models of the MM BM TME

Increased availability of high-powered computers has revolutionized the use of computational, or in silico, modeling in cancer research [125]. In MM, efforts have largely focused on bioinformatic analyses of large genomic and transcriptomic datasets, as well as pharmacokinetic and pharmacodynamic simulations of drug interactions. These approaches have led to key discoveries, including novel signaling pathways [126,127], genetic risk factors [128,129], biomarkers [130,131], RNA interactions [132,133,134,135], and insights into genomic and transcriptional changes [136], and oncogenic inflammation and the BM TME [137,138]. Large datasets have also been leveraged to model the efficacy and side effects of drugs by simulating the interactions between different cell types [137,139]. While these studies primarily apply mathematical modeling to experimental data, one of the more powerful applications of computational modeling is the creation of virtual three-dimensional (3D) models of the BM TME [140], which has enabled visualization of changes in cell behavior and interactions in response to biochemical variations. Agent-based computational models are also emerging where spatial transcriptomics data enable cell simulations, considering each cell’s individual interactions (cell or ECM) within the MM BM TME, providing predictions of cell motility, proliferation, differentiation, apoptosis, and interactions between cells [141]. Single-cell transcriptomics from BM biopsies have revealed gene signatures associated with outcome and resistance signatures acquired in response to therapy, e.g., MYC regulation is associated with acquired resistance to daratumumab [142]. The integration of spatial transcriptomics of MM niches within BM trephines has uncovered composition of surrounding cells that can predict PFS, e.g., CD4 T cells, [143] and the heterogenous nature of the BM TME supporting distinct niches that are different within and between patients [144]. Machine learning models, such as MoSiacNet enable the trephine BM architecture to be characterized and location of immune T cells and MM-PCs mapped [145], along with changes during progression and treatment. Despite their potential, computational models are limited by the quality of the input data and the often-simplified representation of in vivo conditions [146]. However, further advances in this field could enable integration with macroscale models of bone lesions, such as vertebral structures, leading to patient-specific predictions regarding MM disease progression [140].

With the rapidly changing therapeutic landscape and constantly emerging datasets, in silico prediction through artificial intelligence (AI) and digital twin technology are emerging as useful tools for personalized medicine in MM [19,147]. AI has potential to inform clinical practice and therapeutic decisions in real time through the integration of patient information including clinical parameters, imaging and multiomic datasets. Digital twin technology can improve upon AI models, as they can inform clinical practice and therapeutic decisions in real time throughout the course of the disease. This enables dynamic changes in MM clonal heterogeneity within MM-PCs and the BM TME to be incorporated into predictions through treatment simulation.

### 7.3. Three-Dimensional In Vitro Models of the MM BM TME

To more accurately model the biophysical and biochemical effects of the BM TME on MM-PCs, 3D in vitro models of the BM TME have been developed [140]. By incorporating the unique cellular and spatial interactions within the BM, 3D models of the MM BM TME can more accurately reflect the complexity and heterogeneity of the disease [87,140]. The aim of these models is to bridge the gap between in vitro and in vivo settings and improve translation of findings into clinical trials [148]. A variety of methodologies have been investigated to generate 3D models of the BM TME, including scaffold-free, scaffold-based (natural or synthetic polymer, hydrogel, decellularized tissue, or bio-composite), bioprinted, bioreactor, and microfluidic-based models [87,148] (Figure 2). The 3D in vitro models of the MM BM TME developed to date are summarized in Table 2, with further discussion of each type of model in the following sections of this review. Nearly all the studies to date have assessed the response of MM-PCs in 3D models of the MM BM TME to clinically available therapies, predominantly PIs, with fewer studies assessing emerging therapies (Table 2). Most of these studies utilize polymer and hydrogel scaffold-based 3D models, but several published studies have used bio-composite scaffold-based, bioprinting, bioreactor, and microfluidic models (Table 3).

#### 7.3.1. Scaffold-Free and Scaffold-Based 3D Models of the BM TME

Three-dimensional models of the BM TME can be broadly divided into scaffold-free and scaffold-based models [140]. Scaffold-free 3D models rely on cell self-assembly, enabling natural interactions, spatial organization, and physiological responses, though they may lack mechanical support and reproducibility [188]. Spheroid/organoid models form through spontaneous cell association in suspension culture and then secrete their own ECM that facilitates structural and functional support features of the BM niche. These models have the advantage that they are scalable for high-throughput screening [185], can support primary MM cells for up to two weeks on in vitro [182] and are amenable to gene editing. However, scaffold-free models have limitations since they lack both vasculature and the MM-PC interaction with the endosteal bone niche [189], which are both key to MM biology, and as such insufficiently capture adhesion, pro-tumorigenic signals and drug responses.

In contrast, scaffold-based models incorporate natural or synthetic biomaterials to support cell growth and interactions (Figure 2) [190]. Biocompatible scaffolds provide structural and mechanical support and can be used to determine the diffusion of oxygen, nutrients and waste [190,191]. Different types of scaffolds offer unique advantages and disadvantages in terms of their biodegradability, bioactivity, architecture, and mechanical properties [192]. Scaffold properties, such as stiffness, pore size, surface topography, and load bearing capability can also be optimized depending on the model requirements and context in which they are used [193]. Additionally, scaffolds can be biologically active, delivering growth factors, hormones, and regulatory compounds to influence cell behavior, including cytokine release or specific gene expression [80,194]. The different scaffolds currently reported in use for in vitro 3D models of the BM TME are described in Table 3.

#### 7.3.2. Reconstructed BM Models

Scaffold-based models are the most extensively studied 3D culture systems of the MM BM TME. Kirshner et al., (2008) developed one of the earliest models, comprised of a fibronectin/collagen-coated surface to represent the endosteal niche, with patient-derived BM MNCs embedded in a Matrigel-fibronectin hydrogel, which they termed the reconstructed BM (rBM) model [149,155]. Supplementation of this model with autologous patient plasma, but not healthy donor plasma, promoted the proliferation of MM-PCs and putative cancer stem cell-like subpopulations associated with drug resistance and relapse [149]. The rBM model has since been modified in several ways, including by Huang et al., (2018), who fixed the MM-PCs and surrounding matrix in formalin for histologic processing and immunocytochemical studies [165]. In comparison to a conventional 2D culture system, this model led to activation of signal transducer and activator of transcription 3 (STAT3)-mediated signaling in the MM-PCs, which promoted their viability and reduced sensitivity to bortezomib [165]. Similarly, Caillot et al., (2020) determined that MM-PCs cultured in the rBM model were less sensitive to both bortezomib and auranofin, a thioredoxin reductase inhibitor with therapeutic potential, compared to cells in monoculture [174]. These studies suggest that by supporting the survival of MM-PCs, the rBM model may more closely mimic the BM TME and its ability to reduce the efficacy of therapeutic strategies that inhibit STAT3 or rely on the production of reactive oxygen species (ROS). These studies led to the commercialization of the rBM model by zPredicta Inc. as a product known as r-Bone™, which incorporates MSCs, hematopoietic cells, and components of the ECM to replicate the architecture of the BM. In a study of 21 MM patients, the r-Bone™ model was found to be 90% accurate in its ability to predict the clinical response of MM patients to a variety of therapeutic regimens [195].

#### 7.3.3. Gel-Based Scaffold Models

In addition to the rBM model, several 3D in vitro models of the MM BM TME have been generated by embedding MM-PCs and MSCs, or primary MNCs from MM patients, in a gel-based polymer scaffold without modeling of the endosteal niche. Matrigel, which is comprised of fibronectin, laminin, and collagen, is the most common scaffold in these models as it closely resembles native ECM [99,166,167,171,173,176,182]. Other polymers that have been used as gel-based scaffolds in the context of the MM BM TME include silk fibroin [99], gelatin [150], alginate [161], fibronectin [173,176], collagen [162,163,176,182,184], agarose [180,181], HA [179,187], and methylcellulose [185]. Several studies have also incorporated gel-based scaffolds into spheroid or organoid 3D models of the MM BM TME by passive encapsulation of cells [79]. In the last two decades, there has been an increasing number of studies in which hydrogels have been used as scaffolds in models of the MM BM TME. Hydrogels are water-based gel scaffolds that are generated by physical, chemical, or radiation-induced cross-linking of hydrophilic polymers [196]. The mechanical and functional properties of synthetic polymers, such as polyethylene glycol (PEG), can be easily modified, whereas natural polymers such as alginate, chitosan, and collagen are more biocompatible and biodegradable, and have inherent biological activity [196]. Hybrid hydrogels, which are comprised of both natural and synthetic polymers, have the properties of both types of polymer and have been demonstrated to more accurately mimic the BM ECM [79].

Narayanan et al., (2014) demonstrated that the stiffness of HA hydrogels impacts the survival of MM cells encapsulated with BM-derived MSCs, with optimal MM cell survival and proliferation observed with medium-stiffness hydrogels compared to low- or high-stiffness variants [154]. Importantly, this is consistent with the effects of ECM stiffness on cancer progression in vivo, as increased levels of stiffness are associated with enhanced cell proliferation and tumor growth [79]. Jakubikova et al., (2016) developed a hydrogel-based 3D model using the synthetic self-assembling peptide hydrogel, PuraMatrix™ in which they cultured BM-derived MNCs from MM patients [160]. That 3D system resulted in increased expression of ECM molecules and cytokines known to promote MM cell survival, including IL-6, IL-8, monocyte chemoattractant protein-1 (MCP-1 or CCL2), VEGF, collagen I, and fibronectin [160]. Comparing the response of MM-PCs cultured in that model to cells in 2D cultures revealed that the 3D model more closely mirrored the clinical response of the patients to the therapy, suggesting the 3D model may represent a tool for developing personalized therapeutic approaches [160]. Waldschmidt et al., (2022) took a different approach, by seeding primary MM-PCs in an agarose-based microwell device (3D CoSeedis™) prior to culture on a BM-derived MSC monolayer [181]. Although the proliferation of MM-PCs in that model was slower than in 2D cultures, their growth continued over a 12-day period, while the cells in 2D cultures were no longer viable after eight days [181]. Similarly, Braham et al., (2018) showed that primary MM-PCs remained viable for up to 28 days when cultured in a Matrigel-based 3D model and that the model could be used to assess the sensitivity of MM-PCs to sustained release liposome-based drug therapies [166,167]. More recently, Khan et al., (2023) addressed the lack of vascularization of these gel-based models by embedding primary MM-PCs along with induced pluripotent stem cells (iPSC), which differentiated into mesenchymal, endothelial, and hematopoietic lineages, into a hydrogel matrix to generate a BM organoid model [182]. That model maintained the viability of the MM-PCs at 90% for at least ten days, whereas cells in 2D cultures were no longer viable after two days [182].

PIs have been widely tested in 3D models of the MM BM TME. MM-PCs cultured in hydrogel-based systems have consistently been shown to be less sensitive to bortezomib than cells cultured in 2D systems. For example, Reagan et al., (2014) used a hydrogel model that supported osteogenic differentiation and showed that MM-PCs in that model were less sensitive to bortezomib-induced apoptosis [99]. Similarly, Jakubikova et al., (2016) incorporated MM-PCs into a 3D Matrigel/collagen matrix and observed a reduction in the cytotoxic effects of both bortezomib and carfilzomib, which was exacerbated by the inclusion of patient-derived stromal cells [160]. Waldschmidt et al., (2022) employed a HA-based hydrogel to create a patient-specific microenvironment that preserved primary MM cell viability and conferred variable effects on the bortezomib sensitivity of the MM-PCs, which emphasized the importance of considering the heterogeneity between patients in the composition of the ECM in the models [181].

The efficacy of IMiDs has also been assessed in 3D MM BM TME models. MM-PCs embedded in a Matrigel/collagen matrix were significantly less sensitive to all three IMiDs compared to 2D culture systems, and the presence of stromal cells further increased their drug resistance. This highlights the role of both cell–matrix and cell–cell interactions in modulating treatment responses [160].

#### 7.3.4. Solid-Based Scaffold Models

Gel-based scaffold models have been instrumental in the development of 3D in vitro models for MM. However, these soft matrices do not recreate the mineralization that is a feature within the BM [99]. Solid scaffold systems represent a complementary approach to gel-based systems, by providing structure for mechanical support and spatial organization. The model developed by Calimeri et al., (2011) involved coating cylinders formed from poly-ɛ-caprolactone polymeric scaffold (PCLS) with BM MSCs or primary BM MNCs [151]. By surgically implanting these PCLS structures subcutaneously into the flank of SCID mice, the authors were able to expand primary MM-PCs in vivo [151]. Building on this concept, Reagan et al., (2014) engineered solid, porous scaffolds from silk fibroin and demonstrated that MSCs cultured in these structures underwent osteogenic differentiation and changes in their microRNA profiles [99]. These polymer-based scaffolds have also proven effective in modeling MM drug responses; MM cell lines and primary cells cultured in these 3D models were significantly less sensitive to bortezomib compared to cells in 2D culture [99]. Similarly, Ji et al., (2016) documented that MM-PCs seeded into a porous 3D polymer scaffold were less sensitive to both lenalidomide and thalidomide than cells from the same patients cultured under 2D conditions, with the effect attributed to downregulation of CRBN-mediated signaling pathways [162].

Alhallak et al., (2021) developed a porous 3D polycaprolactone scaffold that mimicked the trabecular region of bones, which they found supported MM cell proliferation and protected the cells from bortezomib-, carfilzomib-, and ixazomib-induced apoptosis [177]. Similarly, Clara-Trujillo et al., (2022) fabricated a 3D polyurethane scaffold, functionalized with ECM proteins, and demonstrated that MM-PCs cultured in that system were significantly less sensitive to bortezomib compared to 2D controls [179]. That model was developed further by Ilic et al., (2024), who incorporated both MM-PCs and BM MSCs into a 3D-printed polymer scaffold, which resulted in an increase in cell adhesion, increased expression of markers associated with drug resistance and a reduction in bortezomib sensitivity [184]. Strategies involving coating surfaces, such as those used by Kirshner et al., (2008) [149] and Huang et al., (2018) [165], have been shown to enhance the biomimicry of polymer systems by incorporating proteins found in the bone matrix, including collagen and fibronectin, which confer protection against bortezomib-induced cytotoxicity.

Recently, García-Briega et al., (2025) used a different approach, which involved the generation of solid microspheres, functionalized with either ECM biomolecules or MSCs [187]. These structures were then co-cultured with MM cell lines; however, no effect on viability, cell cycle, or proliferation of the MM-PCs was observed with that approach.

#### 7.3.5. Bio-Composite or Tissue-Engineered Models

Apart from two studies by de la Puente et al., (2015), there have been few investigations of bio-composite or tissue-engineered models of the MM BM TME [156,157]. The authors developed a 3D tissue-engineered BM model, called 3DTEBM^®^, which consisted of a mixture of MM-PCs, ECs, and MSCs from MM patients [156]. The model also included fibrinogen and calcium chloride to promote clotting and cross-linking, with the addition of tranexamic acid to stabilize the structure for up to seven days [156]. Proliferation of MM-PCs co-cultured with MSCs and ECs in that model increased by 250%, compared to cells in monoculture. The 3DTEBM^®^ model was also associated with similar drug and oxygen gradients as those observed in vivo, with higher proliferation rates and greater drug sensitivity observed in MM-PCs that were in proximity to the vasculature [156]. That study and the model developed highlight the importance of in vitro 3D environments that mimic the BM architecture for assessing drug efficacy. In a subsequent study, the 3DTEBM^®^ model was used to assess the efficacy of an antibody targeting CD47 on MM-PCs. The study found the antibody had significant cytotoxic effects against MM-PCs in the 3D model when macrophages were present but no effects under more conventional 2D culture conditions [197]. Similarly, more advanced systems also emphasize the importance of the 3D niche in determining the sensitivity of MM-PCs to IMiDs. Alhallak et al., (2021) employed a bone-mimetic, polycaprolactone scaffold and observed that lenalidomide and pomalidomide treatment had minimal effects on the viability of MM-PCs, particularly when MSCs were present [177]. The accuracy of the 3DTEBM^®^ model at predicting treatment responses in MM patients was impressive, with an 89% concordance between the in vitro results and the clinical outcome of the 19 patients studied [177]. These results demonstrate the potential of the 3DTEBM^®^ model in the context of preclinical testing of novel therapeutic approaches for BM malignancies, including MM.

#### 7.3.6. Bioprinted Models

Bioprinting refers to an emerging technology that has the potential to create models of the MM BM TME through the precise and controlled deposition of cells and biomaterials. Bioprinting enables integration of vascular structures and patient-derived cells into models, which are crucial for studying personalized treatment strategies and the role of angiogenesis in diseases, including MM. Wu et al., (2022) successfully developed a 3D-bioprinted model that recreated the structural and cellular complexity of the MM BM niche by combining MM and stromal cells within a gelatin-based matrix, [175]. The model was used to study tumor–stromal cell interactions and drug responses and revealed spatial heterogeneity in bortezomib responses and greater resistance than observed in a 2D model [175]. In a similar study, Jakubikova et al., (2016) observed significant differences in the behavior and drug sensitivity of primary MM-PCs in a 3D-bioprinted model of the BM niche, compared to cells from the same patients cultured under conventional monoculture conditions [160]. Similarly, bioprinted BM constructs generated in the study by Alhallak et al., (2021) were used to assess patient-specific drug responses, again highlighting the potential of this approach as a tool for predicting the response of individual patients to a particular therapy [177]. These more advanced dynamic and spatially organized systems have also been employed to assess the efficacy of PIs. In their study, Braham et al., (2018) incorporated ECs into a bioprinted model, which enabled an investigation of the roles of the endosteal and perivascular BM regions [168]. That approach permitted co-localization of MM and ECs and demonstrated the capacity of vascular niches to shield MM-PCs from bortezomib and carfilzomib [168]. This is particularly relevant in the context of MM as differences in the cell-to-cell interactions and conditions experienced by MM-PCs in the different BM compartments may have significant effects on the ability of the tumor cells to evade therapies.

In a bioprinted tri-culture model, incorporating MM, MSC, and EC cells, Braham et al., (2018) [168] and Maaike et al., (2019) [169] showed that lenalidomide, thalidomide, and pomalidomide had limited cytotoxicity, particularly in vascularized regions, suggesting that EC support may further diminish the efficacy of IMiDs. Visconti et al., (2021) developed a bio-composite scaffold replicating the osteogenic BM niche and demonstrated that the activity of lenalidomide was significantly altered in the presence of differentiating OBs, suggesting bone-derived cues may impact the sensitivity of MM-PCs to certain therapies [178].

#### 7.3.7. Bioreactor Models

Models known as bioreactors resemble the in vivo BM microenvironment by incorporating dynamic aspects of the tissue, including the mechanical forces associated with fluid flow and changes in the levels of oxygen. Ferrarini et al., (2013) established a 3D MM culture model using the rotary cell culture system (RCCS) bioreactor and demonstrated that the 3D tissue architecture of MM tissue explants was preserved and bortezomib treatment could be assessed [153]. Bioreactor systems developed by Ferrarini et al., (2013) [153], Belloni et al., (2018) [170], and Bonomi et al., (2017) [164] enabled the effects of perfusion and mechanical stimulation to be modelled. The study concluded that these features of the model and the BM TME were likely to reduce the sensitivity of MM-PCs to bortezomib and highlight the potential importance of biomechanics in the progression and treatment responsiveness of MM patients. Silva et al., (2017) also developed an ex vivo system for predicting clinical responses in MM patients involving a bioreactor, which again was proposed as a tool for the development of precision medicines [198]. The dynamic nature of bioreactor-based models means they may represent a versatile resource for MM research, enabling modeling of the effects of biomechanics on the complex cell-to-cell interactions involving tumor cells.

#### 7.3.8. Microfluidic Models

Microfluidic models, also known as “organ-on-a-chip” systems, are similar to bioreactors in that they enable precise control of the cellular microenvironment for studies of the pathobiology of the MM BM TME. However, these models incorporate microscale fluidic channels that simulate the microvasculature within the BM niche. Zhang et al., (2013) developed one of the first patient-specific 3D microfluidic tissue models for MM, which enabled real-time monitoring of the proliferation and drug response of tumor cells [159]. More recent advances in microfluidic technologies have enabled the integration of multiple cell types, including MM-PCs, MSCs, and immune cells, within a single device. Ghoshal et al., (2025) created a multi-niche human BM-on-a-chip platform to study the efficacy of CAR T-cell therapies under dynamic conditions [186], which the authors argued provided a more accurate assessment of the likely in vivo efficacy of the therapy.

Another innovative approach developed by Luanpitpong et al., (2025), involved a high-throughput microfluidic system for assessing the interaction between MM and stroma cells and the drug sensitivity of cancer cells [185]. That model enabled multiple therapeutic agents to be screened in parallel, thereby improving the efficiency of drug discovery. The relatively short time required to establish microfluidic models mean they may be particularly valuable for considering the heterogeneity in drug response between patients. This was demonstrated in a study by Maaike et al., (2019), where a 3D BM microfluidic system was employed to investigate differences in the response of MM-PCs to various treatments [169]. Similarly, Daniela et al., (2018) developed a microfluidic system to study the interaction of MM-PCs with other cells in the BM and their response to multiple drugs, with the findings of the study again highlighting the potential application of microfluidic platforms in preclinical research [170]. Generation of quantitative measurements from microfluidic models that are difficult to obtain from conventional static cultures could enable determination of drug delivery throughout the BM TME under differing flow rates or drug concentrations. Microfluidic platforms developed in the studies by Khin et al., (2014) [152] and Silva et al., (2015) [163] enabled precise control over the position of the cells and drug exposure, revealing that bortezomib efficacy was likely influenced by spatial constraints and gradients within the BM niche. This exemplifies how microfluidic platforms can model spatial pharmacokinetic dynamics or drug accumulation, effects which are absent in conventional static pharmacokinetic models. Control over the composition of microenvironmental interactions and drug concentrations enables characterization of how MM-PC clones evolve under therapeutic pressure through assessment of cellular movement, MM-PC survival within niches and emergence of resistance. However, it is important to note that these models generally consist of a limited immune repertoire and a simplified vasculature, with short experimentation times. Collectively, the studies demonstrate that microfluidic models have significant potential for investigating the role of the BM TME in the pathobiology of MM and for the preclinical evaluation of novel therapeutic strategies, particularly in the context of personalized medicine for MM patients. For organ-on-a-chip technologies to transition from academic to diagnostic or regulatory settings would require a standardized “chip” that could be generated reproducibly at scale and that would demonstrate biological and clinical predictability.

## 8. Conclusions and Future Directions

Despite therapeutic advances, MM remains an incurable disease with an urgent need for new prognostic and therapeutic biomarkers to improve patient outcomes. Given the profound effects of the BM TME on the survival, proliferation and drug sensitivity of MM-PCs, it is essential that novel therapeutic approaches are evaluated in preclinical models that accurately recapitulate the biology of the BM niche that harbors MM-PCs. As highlighted throughout this review, advances in 3D models of the BM, including scaffold-free and scaffold-based models, tissue-engineered systems, bioprinted models, bioreactors, and microfluidic platforms, have enabled increasingly sophisticated modelling of the MM BM TME. These models recapitulate key cellular, biochemical, and biophysical features of MM biology and provide clinically relevant information concerning the sensitivity (and resistance) of MM patients to current (and emerging) treatment options. However, significant challenges remain before these platforms can be adopted as standardized preclinical tools or potential alternatives to animal models.

A major limitation is the lack of standardization across model systems, including variability in biomaterial composition, scaffold architecture, incorporated cell populations, culture conditions, and analytical endpoints. Furthermore, while many studies report enhanced physiological relevance compared with conventional 2D cultures, direct comparisons between model platforms remain limited. Determining how accurately the physical properties of these models, including stiffness and porosity, recapitulate inherently heterogenous healthy and diseased human BM remains challenging. Future studies should prioritize direct cross-comparisons of 3D models using consistent physical and biological parameters, alongside benchmarking against patient-derived BM samples.

The field would also benefit from the establishment of standardized analytical and validation frameworks to improve reproducibility and demonstrate the utility of these models for therapeutic screening. This includes defining benchmarks for assay screening robustness (Z’ > 0.5), reproducibility, and predictive performance. Importantly they also require biological validation to ascertain how faithfully relevant disease features are recapitulated, including their concordance with clinical drug responses and the ability to predict the emergence of resistant clones. Such efforts will be critical for assessing whether 3D MM BM TME models can reliably predict drug efficacy, toxicity, and mechanisms of resistance and ultimately support their broader adoption in preclinical research and drug development.

Collectively, continued refinement, standardization, and validation of advanced MM BM TME models will be essential to fully realize their potential as clinically relevant platforms for studying MM biology and accelerating therapeutic discovery.

## Figures and Tables

**Table 1:** Summary of FDA-approved therapies for multiple myeloma to date.

Therapy	Indication	Target
**Immunomodulatory Drugs (IMiDs)**		
Thalidomide	Approved for use with dexamethasone for all disease stages, including as maintenance therapy without dexamethasone.	CRBN
Lenalidomide	Approved for use for all disease stages, including as maintenance therapy without dexamethasone.	CRBN
Pomalidomide	Approved for use with dexamethasone for RRMM patients who have relapsed after at least 2 prior therapies, including lenalidomide and a PI.	CRBN
**Proteasome Inhibitors (PIs)**		
Bortezomib	Approved for NDMM and for retreatment of patients who had previously responded but relapsed at least 6 months after treatment completion.	Proteasome
Carfilzomib	Approved as a single agent for RRMM patients who have either progressed on or within 60 days of completing a line of therapy with bortezomib and an IMiD or who have progressed after receiving a line of therapy in combination with dexamethasone.	Proteasome
Ixazomib	Approved in combination with lenalidomide/dexamethasone for patients who have received at least 1 prior therapy.	Proteasome
**Monoclonal Antibodies (mAbs)**		
Daratumumab	IV administration approved in combination with lenalidomide and dexamethasone in ASCT-ineligible NDMM patients and RRMM patients who have received at least 1 prior therapy; in combination with VMP in ASCT-ineligible NDMM patients; in combination with VTd in ASCT-eligible NDMM patients; in combination with bortezomib and dexamethasone in patients who have received at least1 prior therapy; in combination with carfilzomib and dexamethasone in RRMM patients who have received 1–3 prior lines of therapy; in combination with pomalidomide and dexamethasone in patients who have received at least 2 prior therapies including lenalidomide and a PI; as monotherapy in patients who have received at least 3 prior lines of therapy, including a PI and an IMiD, or who are double-refractory to a PI and an IMiD. Injection administration approved for use in combination with lenalidomide or VMP for ASCT-ineligible NDMM patients; in combination with VTd in ASCT-eligible patients; in combination with bortezomib and dexamethasone in patients who have received at least 1 prior therapy; in combination with pomalidomide and dexamethasone in patients who have received at least 1 prior line of therapy including lenalidomide and a PI; in combination with carfilzomib and dexamethasone in RRMM patients who have received 1–3 prior lines of therapy; as monotherapy for patients who have received 3 or more prior lines of therapy including a PI or an IMiD or who are double-refractory to a PI and an IMiD.	CD38
Elotuzumab	Approved in combination with lenalidomide/dexamethasone for patients who have received at least 1 prior therapy; or in combination with pomalidomide/dexamethasone for patients who have received at least 2 prior therapies including lenalidomide and a PI.	SLAMF7
Isatuximab	Approved in combination with pomalidomide/ dexamethasone for patients who have received at least 2 prior therapies including lenalidomide and a PI; or in combination with carfilzomib/dexamethasone for RRMM patients.	CD38
**Bispecific Antibodies (BiSAbs)**		
Teclistamab	Approved for the treatment of RRMM patients who have received 4 or more prior lines of therapy, including an IMiD, a PI, and an anti-CD38 mAb.	BCMA
Talquetamab	Approved for the treatment of RRMM patients who have received 4 or more prior lines of therapy, including an IMiD, a PI, and an anti-CD38 mAb.	GPRC5D
Elranatamab	Approved for the treatment of RRMM patients who have received 4 or more prior lines of therapy, including an IMiD, a PI, and an anti-CD38 mAb.	BCMA
**Alkylators**		
Melphalan	Approved for use as a single agent for all disease stages.	DNA fragmentation and damage
Cyclophosphamide	Approved for use as a single agent or in combination with other agents for all disease stages.	
Bendamustine	Approved in Europe for use in combination with prednisone for NDMM patients older than 65 years who are not eligible for ASCT and who have peripheral neuropathy.	
**Anthracyclines**		
Doxorubicin	Approved for use in combination with bortezomib for patients who have not previously received bortezomib and who have received at least 1 prior therapy.	DNA intercalation, inhibition of topoisomerase II, generation of free radicals and oxidative stress
**Corticosteroids**		
Dexamethasone	Approved for use as a single agent, or in combination with other agents, for NDMM and RRMM.	GR
Prednisone	Approved for use as a single agent, or in combination with other agents, for NDMM and RRMM.	GR
**Chimeric Antigen Receptor (CAR) T-Cells**		
Idecaptagene vicleucel (Ide-Cel)	Approved for the treatment of RRMM patients who have received 2 or more prior lines of therapy, including an IMiD, a PI, and an anti-CD38 mAb.	BCMA
Ciltacaptagene autoleucel (Cilta-Cel)	Approved for the treatment of RRMM patients who are refractory to lenalidomide and have received 1 or more prior lines of therapy, including a PI, and an anti-CD38 mAb.	BCMA
**Small-Molecule Inhibitors**		
Selinexor	Approved for use in combination with bortezomib and dexamethasone for patients who have received at least 1 prior therapy; or in combination with dexamethasone for RRMM patients who have received at least 4 prior therapies and whose disease is refractory to at least 2 PIs, at least 2 IMiD, and an anti-CD38 mAb.	XPO1 (Nuclear export inhibitor)
**Antibody–drug Conjugates**		
Belantamab mafodotin-blmf	Approved for treating RRMM in adults that have had at least two prior therapies, in combination with bortezomib and dexamethasone (BVd)	BCMA

Abbreviations: Antibody–drug conjugates (ADCs), autologous stem cell transplant (ASCT), B-cell maturation antigen (BCMA), bispecific antibodies (BiSAbs), bortezomib and dexamethasone (BVd), cluster of differentiation 38 (CD38), cereblon (CRBN), G-protein-coupled receptor, class C, group 5, member D (GPRC5D), glucocorticoid receptor (GR), immunomodulatory drugs (IMiDs), monoclonal antibodies (mAbs), newly diagnosed multiple myeloma (NDMM), proteasome inhibitors (PIs), relapse/refractory multiple myeloma (RRMM), SLAM Family Member 7 (SLAMF7), bortezomib, melphalan, and prednisone (VMP), bortezomib, thalidomide and dexamethasone (VTd), and exportin 1 (XPO1).

**Table 2:** Details of 3D in vitro models of the MM BM tumor microenvironment developed to date.

Year	Reference	Type of Model	Model Composition	Cell Types Incorporated	Drugs Tested
2008	[149]	Coating + Polymer Scaffold	Plates coated with fibronectin/collagen prior to the addition of MNCs suspended in a Matrigel/fibronectin matrix	Primary MSCs, primary MNCs	Chemotherapy (melphalan), proteasome inhibitor (bortezomib)
2009	[150]	Polymer Scaffold	MSCs embedded in gelatin sponge discs	Primary MSCs, MM cell line (RPMI-8226)	None
2011	[151]	Polymer Scaffold/ Animal	Co-cultures embedded in PCLS scaffold cylinders, surgically implanted subcutaneously into the flank of SCID mice	MSC cell line (OP9) or primary MSCs, MM cell line (INA-6) or primary CD138+ MM-PCs	Chemotherapy (dexamethasone), proteasome inhibitor (bortezomib)
2013	[152]	Microfluidic	Co-cultures loaded into 3D cell culture slides with bovine collagen type I	Primary MSCs, primary CD138+ MM-PCs. Fibroblast cell line (HS-5), MM cell lines (RPMI-8226, NCI-H929, 8226/LR-5)	Chemotherapy (melphalan), proteasome inhibitor (bortezomib)
2013	[153]	Bioreactor	Dynamic cultures in RCCS bioreactor	Patient-derived tissue explants (from vertebroplasty, skull, or skin)	Proteasome inhibitor (bortezomib)
2014	[99]	Polymer Scaffold	MSCs embedded in cylindrical, porous, and aqueous silk fibroin scaffolds	Primary MSCs (healthy or patient-derived), MM cell lines (MM.1S, OPM2) or primary CD138+ MM-PCs	Proteasome inhibitor (bortezomib)
2014	[99]	Hydrogel Scaffold	Co-cultures embedded in a fibrinogen/thrombin/Matrigel hydrogel	Primary MSCs (healthy or patient-derived), MM cell lines (MM.1S, OPM2) or primary CD138+ MM-PCs	Proteasome inhibitor (bortezomib)
2014	[154]	Hydrogel Scaffold	Primary MNCs embedded in a Me-HA/Me-Gel hydrogel	Primary MSCs or primary MNCs	None
2014	[155]	Coating + Polymer Scaffold	MNCs embedded in a Matrigel/ fibronectin matrix added to plates coated with fibronectin/collagen	Primary MSCs and primary MNCs	None
2015	[156,157]	Bio-composite Scaffold	Co-cultured cells added to a scaffold formed by cross-linking plasma fibrinogen with calcium chloride and tranexamic acid	Primary HUVECs, MSCs, MM cell lines (MM1, H929, RPMI-8226) or primary CD138+ MM-PCs	PIs (bortezomib, carfilzomib)
2015	[158]	Spheroid/ Animal	Spheroid grafted on chorioallantoic membrane of chicken embryos	Primary MSCs, MM cell lines (OPM-2, RPMI-8226)	Proteasome inhibitor (bortezomib)
2015	[100,159]	Microfluidic	Human fibronectin-coated PDMS microfluidic chambers	OB cell line (hFOB), primary MNCs	None
2016	[160]	Hydrogel Scaffold	MSCs embedded in a PuraMatrix hydrogel	Primary MSCs, and MM cell lines (OPM1, KMS11, OCIMY5) or primary CD138+ MM-PCs	IMiDs (thalidomide, lenalidomide, pomalidomide), PIs (bortezomib, carfilzomib), Chemotherapy (doxorubicin, dexamethasone, melphalan)
2017	[161]	Polymer Scaffold	3D tumor spheroids generated from cells embedded in alginate beads	MM cell lines (NCI-H929, RPMI 8226, OPM-2, JJN-3, U266), primary plasma cell leukemia cells	HDAC inhibitor (SAHA), TRAIL
2017	[162]	Polymer Scaffold	MM-PCs embedded in collagen gel	MM cell line (U266)	IMiDs (thalidomide, lenalidomide), proteasome inhibitor (bortezomib)
2017	[163]	Microfluidic	Co-cultures embedded in a collagen matrix	Primary MSCs, MM cell line (MM.1S) or primary CD138+ MM cell	Chemotherapy (melphalan), proteasome inhibitor (bortezomib)
2017	[164]	Bioreactor	Dynamic cultures in RCCS bioreactor	Primary MSCs, MM cell line (RPMI-8226)	Chemotherapy (paclitaxel), proteasome inhibitor (bortezomib)
2018	[165]	Coating + Polymer Scaffold	MM-PCs embedded in a Matrigel/ fibronectin/collagen matrix added to plates coated with fibronectin/collagen	MM cell lines (U266, RPMI-8226)	STAT3 Inhibitor (stattic), proteasome inhibitor (bortezomib)
2018	[166,167]	Polymer Scaffold	Co-cultures embedded in Matrigel	Primary MSCs, EPCs, and MM cell lines (OPM2, U266, L363) or primary CD138+ MM-PCs	TEGs (αβ T-cells engineered to express a defined γδTCR)
2018	[166,167]	Polymer Scaffold	Co-cultures embedded in Matrigel	Primary MSCs, EPCs, and MM cell lines (OPM2, U266, L363) or primary CD138+ MM-PCs	Untargeted and integrin α4β1 (very late antigen-4) targeted liposomes, liposome-encapsulated chemotherapy (doxorubicin) and proteasome inhibitor (bortezomib)
2018	[168,169]	Bioprinting	Bioprinted two-compartment BM model with either MSCs or EPCs (Matrigel/ CPC)	Primary MSCs, EPCs, MM cell lines (OPM2, L363) or primary CD138+ MM-PCs	IMiDs (thalidomide, lenalidomide, pomalidomide), PIs (bortezomib, carfilzomib), chemotherapy (melphalan, 4-hydroperoxy-cyclophosphamide)
2018	[170]	Bioreactor	Dynamic cultures in RCCS bioreactor	Fibroblast cell line (HS-5) or murine L-fibroblasts, MM cell lines (MM1.S, U266, RPMI-8226). Primary MSCs or MSC-derived OBs, HUVECs, primary CD138+ MM-PCs	Chemotherapy (dexamethasone, melphalan), proteasome inhibitor (bortezomib), monoclonal antibody (natalizumab (anti-VLA-4))
2019	[171]	Polymer Scaffold	Co-culture spheroids suspended in Matrigel	MM cell lines (U266, OPM2, MM,1S), and primary CD14+ monocytes	NER-targeting agent (trabectedin), ascorbic acid
2020	[172]	Hydrogel Scaffold	MSCs embedded in a 10% w/v PGMA_52_-PHPMA_122_ copolymer worm hydrogel spheres	Primary MSCs, MM cell lines (RPMI-8226, MM.1S, JJN3)	None
2020	[173]	Polymer Scaffold	MNCs embedded in a Matrigel/ fibronectin matrix and overlaid with patient-derived plasma	Primary MNCs	Monoclonal antibody (daratumumab (anti-CD38))
2020	[174]	Coating + Polymer Scaffold	MM-PCs embedded in a Matrigel/ fibronectin/collagen matrix added to plates coated with fibronectin/collagen	MM cell lines (H929, L363) or primary MNCs	NOX inhibitor (VAS3947), proteasome inhibitor (bortezomib), antioxidant (NAC), V-ATPase inhibitor (BafA1), thioredoxin reductase inhibitor (auranofin)
2021	[175]	Bioprinting	Fibroblasts and MM-PCs mixed with core bioink (GelMA, alginate, PEGDA, nHA) and printed into tubular structures	Fibroblast cell line (HS-5), MM cell lines (MM.1S, RPMI-8226) or primary CD138+ MM-PCs	Monoclonal antibody (tocilizumab (anti-IL-6R)), proteasome inhibitor (bortezomib)
2021	[176]	Polymer Scaffold	MNCs embedded in a Matrigel/ fibronectin/collagen matrix	Primary MNCs	STAT3 inhibitor (Stattic), IL-6
2021	[177]	Polymer Scaffold	Co-cultured cells added to a scaffold formed by cross-linking CaCl_2_ and tranexamic acid	MM cell lines (MM.1S, H929, OPM2) or primary CD138+ MM-PCs	Monoclonal antibody (daratumumab (anti-CD38)), IMiDs (lenalidomide, pomalidomide), PIs (bortezomib, carfilzomib, ixazomib), Chemotherapy (doxorubicin, dexamethasone, melphalan, etoposide), HDAC inhibitor (panobinostat)
2021	[178]	Bio-composite Scaffold	OBs embedded in a Matrigel matrix	Primary MSC-derived OBs, MM cell lines (MM.1S, NCI-H929, U266 RPMI-8226)	IMiD (lenalidomide), bisphosphonate (alendronate), monoclonal antibodies (anti-sclerostin, anti-DKK1)
2022	[179]	Polymer Scaffold	MM-PCs embedded in fibronectin or hyaluronic acid microspheres	MM cell lines (RPMI-8226, U226, MM.1S)	Chemotherapy (dexamethasone), proteasome inhibitor (bortezomib)
2022	[180,181]	Hydrogel Scaffold	Disk-shaped agarose device featuring conical microwells	Fibroblast cell line (HS-5), MM cell lines (RPMI-8226, U266, OPM-2) or primary CD117+ MM-PCs	Proteasome inhibitor (bortezomib), thioredoxin reductase inhibitor (auranofin), KDAC inhibitors (JS08, JS28, JS46, entinostat, panobinostat, vorinostat), KDM inhibitors (SYN22327689, Z425307602 and KDM5-C49)
2022	[180,181]	Hydrogel Scaffold	Disk-shaped agarose device featuring conical microwells	Fibroblast cell line (HS-5), MM cell lines (RPMI-8226, U266, OPM-2) or primary CD117+ MM-PCs	Proteasome inhibitor (bortezomib), thioredoxin reductase inhibitor (auranofin)
2023	[182]	Hydrogel Scaffold	iPSCs embedded in a collagen/Matrigel hydrogel matrix	Primary MSCs and primary CD138+ MM-PCs	TGF-β
2024	[183]	Bioreactor	Dynamic cultures in RCCS bioreactor	Fibroblast cell line (HS-5), MM cell lines (MM.1S, U266, RPMI-8226, OPM2)	Cytokines/chemokines: TNF-α, IL-6, IL-1β, SDF-1
2024	[184]	Polymer Scaffold	Co-cultures encapsulated in a collagen matrix isolated from rat tail fibers	Primary MSCs, MM cell line (MM.1S)	Proteasome inhibitor (bortezomib)
2025	[185]	Polymer Scaffold	Co-cultured cells embedded in a methylcellulose matrix	Fibroblast cell line (WI-38–40) or MSC cell line (UE6ET-17), MM cell lines (NCI-H929, MM.1R)	None
2025	[186]	Microfluidics	3D micro-vascularized culture system modeling the endosteal and perivascular BM niches	Primary MSCs, MSC-derived OBs, HUVECs, NHLF-derived pericytes and EPCs, MM cell lines (KMS18, OCIMY5, OPM2, RPMI-8226, U266) or primary CD138+ MM-PCs	CAR-T
2025	[187]	Polymer Scaffold	MM-PCs mixed with microsphere generated with HS, CS, HA	Primary MSCs, MM cell lines (RPMI-8226, U266, MM.1S)	None

Abbreviations: Bafilomycin A1 (BafA1), chondroitin sulphate (CS), endothelial progenitor cell (EPC), hyaluronic acid (HA), histone deacetylase (HDAC), heparan sulphate (HS), human umbilical vein endothelial cells (HUVEC), lysine deacetylase (KDAC), JARID1-selective lysine demethylase inhibitors (KDM), induced pluripotent stem cells (iPSCs), methacrylated hyaluronic acid (Me-HA), methacrylated gelatin (Me-Gel), multiple myeloma (MM), mononuclear cell (MNC), mesenchymal stem cell (MSC), osteoblast (OB), N-acetyl-l-cysteine (NAC), nucleotide excision repair (NER), NADPH oxidase (NOX), poly-ɛ-caprolactone polymeric scaffold (PCLS), polydimethylsiloxane (PDMS), proteasome inhibitors (PIs), rotary cell culture system (RCCS), signal transducer and activator of transcription 3 (STAT3), stromal cell-derived factor 1 (SDF-1), transforming growth factor beta (TGF-β), tumor necrosis factor (TNF-α), tumor necrosis factor-related apoptosis-inducing ligand (TRAIL), and vacuolar H + -ATPase (V-ATPase).

**Table 3:** Details of the scaffolds used for 3D in vitro models of the BM tumor microenvironment.

Scaffold	Description	Biomaterial Components
**Natural Polymers**	Derived from natural sources, these scaffolds mimic the ECM and support cell growth.	Silk fibroin, collagen, chitosan, alginate, fibrin, hyaluronic acid, bacterial nanocellulose, fibronectin, Matrigel, agarose
**Synthetic Polymers**	Made from synthetic biodegradable materials, these scaffolds offer precise, customizable properties	Poly(ethylene) glycol (PEG), poly(ε-caprolactone) (PCL), Poly(amino acid)-based polymers, poly (DL-lactide-co-glycolide) (PLG)
**Hydrogels**	A 3D hydrophilic polymer network that retains water, creating a soft, hydrated environment for cell growth	Generated using the natural or synthetic polymers listed above, either alone or in combination
**Decellularized Scaffolds**	Removes cells from native tissues, preserving the ECM and bioactive molecules to create a scaffold that mimics the natural tissue environment	Generated from autogenous, allogeneic, or xenogeneic tissues
**Bio-composite Scaffolds**	Hybrid structures combining natural and synthetic materials to enhance biocompatibility, mechanical strength, and bioactivity	Any combination of the biomaterials listed above

## Data Availability

No new data were created or analyzed in this study.

## Abbreviations

2D, two-dimensional; 3D, three-dimensional; ADCs, antibody–drug conjugates; ADCC, antibody-dependent cell-mediated cytotoxicity; ADCP, antibody-dependent cellular phagocytosis; AKT, protein kinase B; ANGPTL4, angiopoietin-like 4; AI, artificial intelligence; ASCT autologous stem cell transplant; BafA1, bafilomycin A1; BAFF, B-cell activating factor; BCL-2, B-cell leukemia/lymphoma 2; BCMA, B-cell maturation antigen; BiSAb, bispecific antibodies; BiTEs, bi-specific T-cell engagers; Blenrep, belantamab mafodotin; BM, bone marrow; BR3, B-cell activating factor receptor; BTK, Bruton’s tyrosine kinase; CAR, chimeric antigen receptor; CAs, chromosomal abnormalities; CD, cluster of differentiation; CDC, complement-dependent cytotoxicity; CELMoDs, cereblon E3 ligase modulators; CHI3L1, chitinase-3-like-1; CRAB, calcium, renal failure, anemia, bone lesions; CRBN, cereblon; CS, chondroitin sulphate; CT, computed tomography; CXCL1, CXC motif chemokine ligand 1; CXCR1/2/4, CXC motif chemokine receptor 1/2/4; DC, dendritic cell; DDB1, DNA damage-binding protein 1; ddPCR, digital droplet polymerase chain reaction; DKK1, Dickkopf-1; DSG2, desmoglein-2; DUSP1, dual-specificity phosphatase; D-VRd, daratumumab in combination with VRd therapy; EC, endothelial cell; ECM, extracellular matrix; EPC, endothelial progenitor cell; ER, endoplasmic reticulum; FcRH5, Fc receptor-homolog 5; FDA, Food and Drug Administration; FISH, fluorescent in situ hybridization; FGF2, fibroblast growth factor 2; GPRC5D, G-protein-coupled receptor, class C, group 5, member D; GR, glucocorticoid receptor; GSI, gamma secretase inhibitor; HA, hyaluronic acid; HDAC, histone deacetylases; HGF, hepatocyte growth factor; HR, high risk; HS, heparan sulphate; HUVEC, human umbilical vein endothelial cells; IKZF3, Aiolos; IL, interleukin; IMiDs, immunomodulatory drugs; iPSCs, induced pluripotent stem cells; Isa-VRd, isatuximab in combination with VRd; ISS, International Staging System; KDAC, lysine deacetylase; KDM, JARID1-selective lysine demethylase inhibitors; KSP, kinesin spindle protein; LAG-3, lymphocyte-activation gene 3, LDH, lactic acid dehydrogenase; mAbs, monoclonal antibodies; MCL-1, myeloid cell leukemia 1; mcMMAF, maleimidocaproyl monomethyl auristatin F; MCP-1, monocytes chemoattractant protein-1; Me-Gel, methacrylated gelatin; Me-HA, methacrylated hyaluronic acid; MGUS, monoclonal gammopathy of undetermined significance; MM, multiple myeloma; MMP, matrix metalloprotease; MNC, mononuclear cell; MRD, minimal residual disease; MRI, magnetic resonance imaging; MSC, mesenchymal stem cell; MUC1, mucin 1; NAC, N-acetyl-l-cysteine; NDMM, newly diagnosed multiple myeloma; NER, nucleotide excision repair; NGF, nerve growth factor; NGS, next-generation sequencing; NK, natural killer; NOX, NADPH oxidase; OB, osteoblast; OC, osteoclast; PCs, plasma cells; PCLS, poly-ɛ-caprolactone polymeric scaffold; PD-1, programmed cell death protein 1; PDMS, polydimethylsiloxane; PEG, polyethylene glycol, PET, positron emission topography; PFS; progression-free survival; PIs, proteasome inhibitors; rBM, reconstituted bone marrow; RCCS, rotary cell culture system; R-ISS, Revised International Staging System; R2-ISS, Second Revised International Staging System; ROS, reactive oxygen species; RRMM, relapse/refractory multiple myeloma; RUNX1, Runt-related transcription factor 1; SDF-1, stromal cell-derived factor 1; sFLC, secreted free light chain; SLAMF7, SLAM Family Member 7; SMM, smoldering multiple myeloma; SR, standard risk; STAT3, signal transducer and activator of transcription 3; TACI, transmembrane activator and CAML interactor; TGF-β, transforming growth factor beta; TME, tumor microenvironment; TNF, tumor necrosis factor; TP53, tumor protein p53; TRAIL, tumor necrosis factor-related apoptosis-inducing ligand; Treg, T regulatory lymphocyte; TriSAbs; tri-specific antibodies; V-ATPase, vacuolar H + -ATPase; VCAM-1, vascular cell adhesion protein 1; VEGF, vascular endothelial growth factor A; VMP, bortezomib, melphalan, and prednisone; VRd, lenalidomide, bortezomib and dexamethasone therapy; VTd, bortezomib, thalidomide, dexamethasone; WEE1, WEE1 G2 checkpoint kinase; XPO1, exportin 1.
